# Network vulnerability-based and knowledge-guided identification of microRNA biomarkers indicating platinum resistance in high-grade serous ovarian cancer

**DOI:** 10.1186/s40169-019-0245-6

**Published:** 2019-10-29

**Authors:** Xin Qi, Chunjiang Yu, Yi Wang, Yuxin Lin, Bairong Shen

**Affiliations:** 1 Center for Systems Biology, Soochow University, Suzhou, 215006 China; 2 School of Nanotechnology, Suzhou Industrial Park Institute of Services Outsourcing, Suzhou, 215006 China; 3 Institutes for Systems Genetics, West China Hospital, Sichuan University, Chengdu, 610041 China

**Keywords:** miRNA biomarker, Platinum resistance, Ovarian cancer, miRNA-mRNA regulatory network, Bioinformatics model

## Abstract

**Background:**

High-grade serous ovarian cancer (HGSC), the most common ovarian carcinoma type, is associated with the highest mortality rate among all gynecological malignancies. As chemoresistance has been demonstrated as the major challenge in improving the prognosis of HGSC patients, we here aimed to identify microRNA (miRNA) biomarkers for predicting platinum resistance and further explore their functions in HGSC.

**Results:**

We developed and applied our network vulnerability-based and knowledge-guided bioinformatics model first time for the study of drug-resistance in cancer. Four miRNA biomarkers (miR-454-3p, miR-98-5p, miR-183-5p and miR-22-3p) were identified with potential in stratifying platinum-sensitive and platinum-resistant HGSC patients and predicting prognostic outcome. Among them, miR-454-3p and miR-183-5p were newly discovered to be closely implicated in platinum resistance in HGSC. Functional analyses highlighted crucial roles of the four miRNA biomarkers in platinum resistance through mediating transcriptional regulation, cell proliferation and apoptosis. Moreover, expression patterns of the miRNA biomarkers were validated in both platinum-sensitive and platinum-resistant ovarian cancer cells.

**Conclusions:**

With bioinformatics modeling and analysis, we identified and confirmed four novel putative miRNA biomarkers, miR-454-3p, miR-98-5p, miR-183-5p and miR-22-3p that could serve as indicators of resistance to platinum-based chemotherapy, thereby contributing to the improvement of chemotherapeutic efficiency and optimization of personalized treatments in HGSC.

## Background

High-grade serous ovarian cancer (HGSC) is the most common histotype accounting for more than 70% deaths from ovarian cancer [1], the leading cause of gynecologic malignancy-induced mortality in women [2, 3]. Due to a lack of early symptoms and effective screening measures, the majority of patients (> 80%) are diagnosed at the advanced stages (FIGO stage III or IV), with 22,440 new cases and 14,080 deaths recorded in the United States in 2017 [2]. Despite improvements in treatment based on optimized surgery techniques and combinational chemotherapy over the past two decades, the overall 5-year survival rate is only 30% in patients with advanced ovarian cancer due to the initial and acquired resistance to platinum-based chemotherapy [4, 5]. Therefore, identifying biomarkers that facilitate detection of patients who may benefit from the platinum-based chemotherapy or otherwise hold significant potential in optimizing personalized therapy, avoiding unnecessary treatment, and eventually improving prognosis of women with ovarian cancer.

MicroRNAs (miRNAs) are approximately 22-nt non-coding RNAs that post-transcriptionally modulate gene expression in a sequence-specific manner via mRNA degradation or translational repression [6]. These molecules are extensively involved in regulation of cellular processes, including proliferation, differentiation, and apoptosis. Moreover, 52.5% human miRNA genes are located in fragile sites or genomic positions associated with cancer [7], suggesting a close association with tumorigenesis. Accumulating evidence has demonstrated that multiple miRNAs are aberrantly expressed in various cancer types and contribute to the initiation and progression of cancer as oncogenes or tumor suppressors [8]. Importantly, these miRNAs can be effectively used as predictive biomarkers or therapeutic targets to optimize cancer diagnosis and treatment regimens [9, 10].

As platinum resistance is a major obstacle in ovarian cancer treatment, extensive efforts have been made to discover miRNA biomarkers involved in drug resistance. For example, Vecchione et al. [11] identified a miRNA signature comprising miR-484, -642, and -217 useful in predicting chemoresistance in ovarian cancer and further demonstrated effects of miR-484 on tumor angiogenesis through regulating the VEGFB and VEGFR2 signaling pathways. Up-regulation of miR-93 was shown to affect cellular response to cisplatin via regulating PTEN/Akt signaling in cisplatin-resistant ovarian cancer cells [12]. miR-141 has been identified as a crucial regulator of cisplatin sensitivity via targeting KEAP1 involved in the NF-κB pathway [13]. However, the majority of studies on platinum resistance to date have primarily focuses on identifying differentially expressed miRNAs followed by molecular mechanism research; therefore, they were unable to consider interaction relationships between different interacting biological players or uncover key regulators at the system level.

Cancer is a complex disease involving dysregulation of multiple signaling pathways. Application of computational approaches to characterize miRNA and/or mRNA signatures from a systems biology perspective has been extensively adopted in various cancers [14]. In our previous studies, miRNA-mRNA network-driven computational methods including Pipeline of Outlier MicroRNA Analysis (POMA) and MicroRNA Biomarker Discovery (MicroRNA-BD), were developed to identify miRNA biomarkers for predicting cancer initiation and metastasis with high performance [15, 16]. Here, we focused on identifying miRNA biomarkers with promising potential in indicating platinum resistance for HGSC patients, thereby facilitating the optimization of personalized therapy. Firstly, we developed a computational method based on miRNA-mRNA regulatory network and single-line regulation theory to identify miRNA biomarkers that may be useful predictors of platinum resistance and prognostic outcomes in HGSC. The functional mechanisms of the identified miRNAs were further investigated by performing GO and pathway enrichment analyses. Importantly, to establish the platinum resistance-associated functions of these miRNAs, their expression patterns were validated in platinum-resistant and -sensitive ovarian cancer cells.

## Methods

### Data collection

To explore miRNA biomarkers associated with platinum-resistance in ovarian cancer, the mRNA and miRNA expression data and clinical data of 31 platinum-sensitive and 37 platinum-resistant HGSC patients were downloaded from the publicly available International Cancer Genome Consortium (ICGC) based on search term ‘OV-AU’, which has been published by Patch et al. [17] in Nature (2015). The miRNA raw data were deposited in the Gene Expression Omnibus (GEO) datasets with the accession number GSE65821, and the transcriptome sequencing raw data were deposited in the European Genome-phenome Archive (EGA) repository under the accession code EGAD00001000877.

To validate the expression pattern of the identified miRNA biomarkers associated with platinum resistance, miRNA expression datasets of platinum-sensitive ovarian cancer cell lines (A2780_S and OVCAR3_S) and corresponding platinum-resistant ovarian cancer cell lines (A2780_R and OVCAR3_R) were downloaded from gene expression omnibus (GEO) under the accession number of GSE84200.

### Identification of differentially expressed mRNAs and miRNAs

The empirical bayes (eBayes) method in ‘limma’ R package [18, 19] was employed to identify differentially expressed mRNAs and miRNAs between platinum-resistant and platinum-sensitive HGSC patients. The Benjamini–Hochberg false discovery rate method was employed to adjust raw P-values. The mRNAs and miRNAs were considered significantly differentially expressed based on the following criteria: fold change > 1.5 or < 0.67 and adjusted P-value < 0.05. Similarly, differentially expressed miRNAs between platinum-resistant and -sensitive ovarian cancer cells (A2780 or OVCAR3) were identified using the same method.

### Construction of the platinum-resistance associated miRNA-mRNA network in HGSC

To investigate the contributory roles of miRNAs in regulating platinum resistance in ovarian cancer, the platinum-resistance associated miRNA-mRNA network in HGSC (PRMNH) was constructed based on the following two steps. Firstly, a human miRNA-mRNA regulatory network was generated using both experimentally validated and computationally predicted miRNA–mRNA regulatory interactions from public databases as used in the miRNA-BD tool [20]. Secondly, differentially expressed mRNAs and miRNAs were mapped onto the human miRNA-mRNA network to extract the PRMNH, which was visualized using Cytoscape 3.6.1 software [21]. Topological property analyses, including degree, betweenness centrality (BC) and closeness centrality (CC), were then performed using the ‘igraph’ package in R. Among them, node degree refers to the number of connecting edges; node betweenness is determined by the number of shortest paths passing the node and represents the role of the node in transactions among other nodes; CC represents the closeness extent of a node to other nodes.

### Identification of platinum resistance-associated miRNA biomarkers

As reported by Lin et al. [16], miRNAs with significantly high number of single-line regulation in the miRNA-mRNA network (NSR) values possess stronger independent regulatory ability, which are vulnerable and important to the stability of biological network. Accordingly, MicroRNA-BD has been developed to discover miRNA biomarkers by considering the single-line regulatory power (measured by NSR) and the tendency to regulate transcription factor gene (measured by TF gene percentage, TFP) [20]. Here, miRNAs with significantly higher NSR values (*P *< 0.01, Wilcoxon signed-rank test) in PRMNH were identified using MicroRNA-BD tool. As a miRNA that regulates platinum resistance-associated target genes is more likely to play key roles in resistance to chemotherapy, the selected miRNAs were further filtered based on the existence of platinum resistance-associated target genes in PRMNH. The functions of miRNA target genes were further searched in the PubMed database. Known platinum resistance-associated miRNAs in ovarian cancer were obtained from the review by Zhang et al. [22] and papers by Wang et al. [23] and Arrighetti et al. [24].

### Receiver operating characteristic (ROC) curve analysis

To evaluate the sensitivity and specificity of identified miRNA biomarkers for distinguishing between platinum-sensitive and platinum-resistant patients, ROC curve analyses were conducted using the ‘ROCR’ package in R [25].

### Survival analysis

To establish the prognostic value of miRNA biomarkers, patients were divided into high-expression and low-expression groups using the upper quartile of miRNA expression level as the threshold. Then, Kaplan–Meier survival analyses were conducted to evaluate the differences in progression free survival (PFS) and overall survival (OS) times between high-expression and low-expression groups by the ‘survival’ package in R, and the log-rank test was employed to assess the statistical significance of the survival curves (*P* < 0.05).

### Functional enrichment analysis

To investigate the functions of the identified miRNA biomarkers, miRNA targets were predicted using Targetscan7.2 [26]. Next, GO enrichment analysis of miRNA target genes was performed using DAVID Bioinformatics Resources version 6.7 [27] and pathway enrichment analysis of those genes was carried out via Ingenuity Pathway Analysis (IPA) [28]. The Benjamini–Hochberg method was applied to adjust raw P-values to the false discovery rate. Adjusted P-values < 0.05 and fold enrichment > 1.5 were used as the cut-off for selecting statistically significant GO and KEGG terms. Enriched GO terms were additionally clustered based on similar functions using the Enrichment Map plugin in Cytoscape 3.6.1 [29].

## Results

### Functional characterization of the platinum resistance-associated miRNA-mRNA network in HGSCs (PRMNH)

**Fig. 1:**
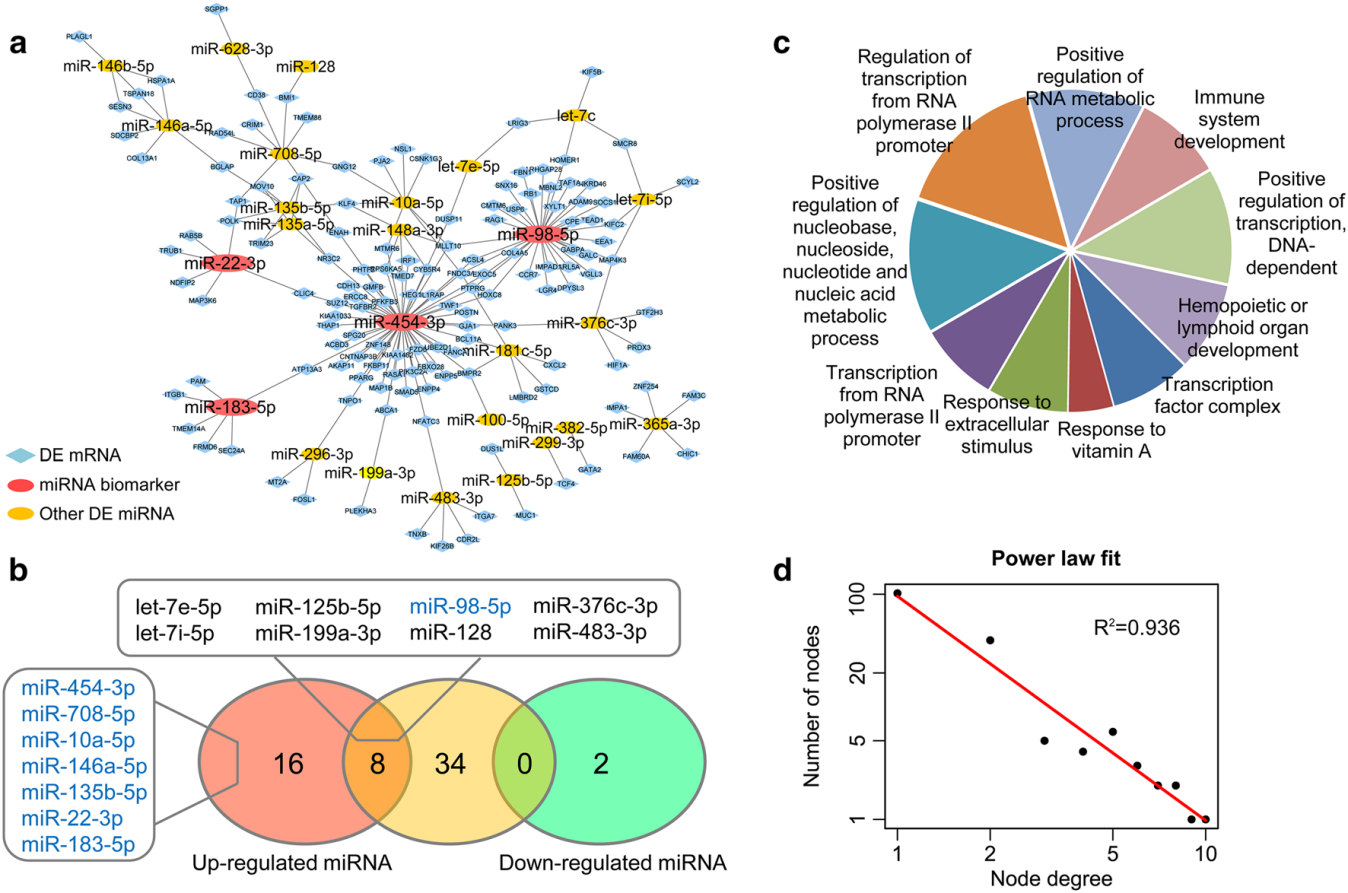
Structural and functional characteristics of PRMNH. **a** Layout of PRMNH consisting of 190 regulations between 26 miRNAs and 140 mRNAs with differential expression patterns. **b** Intersection between known platinum resistance-associated miRNA biomarkers and up- or down-regulated miRNAs in PRMNH. The light red and green ellipses represent up-regulated and down-regulated miRNAs in PRMNH, respectively; light yellow ellipses represent known miRNA biomarkers involved in platinum resistance. miRNAs with properties of both hub and bottleneck nodes were highlighted with blue color. **c** Top 10 significantly enriched GO terms at the biological process level of miRNA target genes in PRMNH. The fan area represents the number of target genes implicated in the corresponding GO term. **d** Degree distribution of all nodes in PRMNH

To systematically explore the miRNA biomarkers associated with resistance to chemotherapy, differential expression analyses were initially performed by comparing the expression profiles of miRNAs and mRNAs between 31 platinum-sensitive and 37 platinum-resistant HGSC patients, respectively. A total of 39 miRNAs (Additional file 1) and 1210 mRNAs (Additional file 2) displayed significantly differential expression pattern. By integrating functional miRNA-mRNA regulatory relationships using MiRNA-BD tool [20], we then constructed PRMNH consisting of 190 miRNA-mRNA regulatory pairs among 26 differentially expressed miRNAs and 140 differentially expressed mRNAs (Fig. 1a, Additional file 3). Among the known platinum resistance-associated miRNAs (Additional file 4) commonly identified in ovarian cancer cell lines, such as A2780, OVCAR3, and SKOV3 [22–24], we recognized 8 up-regulated miRNAs involved in regulating platinum resistance in PRMNH, while 18 other differentially expressed miRNAs (16 up-regulated and 2 down-regulated miRNAs) were detected for the first time (Fig. 1b). The comparing result suggests that this study could provide more insights into the mechanism of platinum resistance in ovarian cancer. To further investigate miRNAs function in PRMNH, GO enrichment analysis was carried out for the target genes of miRNAs in PRMNH. As shown in Fig. 1c, the differentially expressed target genes of miRNAs in PRMNH mainly participated in transcriptional regulation, response to extracellular stimulus, and regulation of nucleic acid metabolic processes, which are closely linked to cancer initiation or progression [30]. Therefore, systematic analysis of the miRNA-mRNA regulatory relationship could provide comprehensive insights into the dysregulated functions of miRNAs involved in platinum resistance in ovarian cancer.

As observed, the degree distribution of all nodes in the PRMNH closely followed a power law distribution with R^2^ = 0.936 (Fig. 1d), indicating that similar to many biological networks, PRMNH was a scale-free network. Accordingly, we investigated the hub and bottleneck nodes in PRMNH, which were typically defined as the top 10% of the highest degree nodes and betweenness centrality nodes, respectively. Strikingly, eight miRNAs (miR-454-3p, miR-98-5p, miR-708-5p, miR-10a-5p, miR-146a-5p, miR-135b-5p, miR-22-3p and miR-183-5p) possessed properties of both hub and bottleneck nodes (Fig. 1b), suggesting significant potential in regulating platinum resistance in HGSC.

### Identifying miRNA biomarkers associated with platinum resistance in HGSC based on miRNA-mRNA regulatory network

**Fig. 2:**
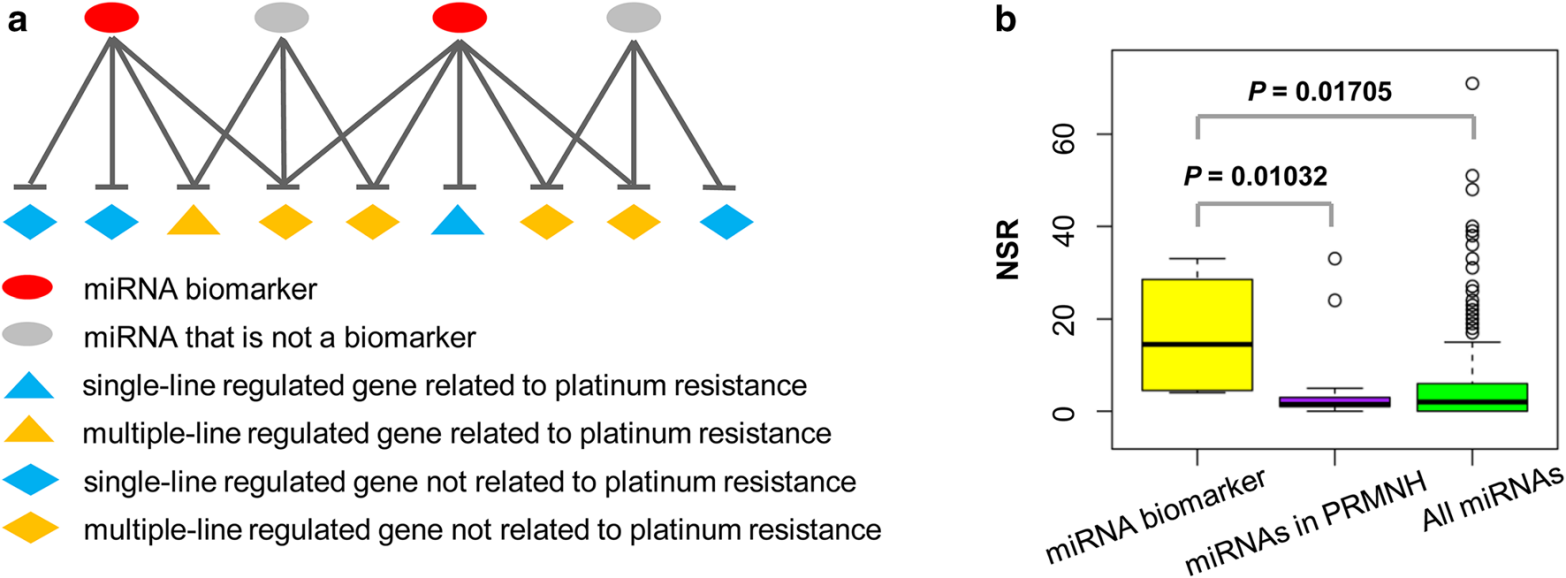
Schematic description of criteria for identifying platinum resistance-associated miRNA biomarkers based on miRNA-mRNA regulatory relationships. **a** Schematic description of miRNA-mRNA regulatory types. **b** NSR distribution of the identified miRNA biomarkers, all miRNAs in PRMNH and all miRNAs in the human miRNA-mRNA regulatory network. Statistical significance was calculated using the Wilcoxon rank-sum test

In PRMNH, target genes can be divided into single-line and multiple-line regulated groups based on the number of miRNAs that regulate the same target gene (Fig. 2a). As reported, miRNA-mRNA pairs with single-line regulated interactions are considered as the vulnerable structure in the network; therefore, they tend to be more important for the stability of biological networks [20]. To date, the strategy of single-line regulation has been successfully applied to discover miRNA biomarkers involved in diagnosis and prognosis of various complex diseases [15, 16, 31, 32]. Considering the key role of platinum resistance-associated genes in chemotherapy response, we adopted two criteria to identify miRNA biomarkers based on PRMNH: (1) miRNAs containing significantly higher NSR value; (2) the presence of miRNA target gene associated with platinum resistance.

As shown in Table 1 and Additional file 5, four miRNAs (miR-454-3p, miR-98-5p, miR-183-5p and miR-22-3p), met the screening criteria and were consequently identified as platinum resistance-associated miRNA biomarkers. These miRNA biomarkers possessed significantly higher NSR values compared with all miRNAs in the PRMNH (*P *= 0.01032, Wilcoxon rank sum test) and all miRNAs in the human miRNA-mRNA network (*P *= 0.01705, Wilcoxon rank sum test) (Fig. 2b). Moreover, the four miRNAs served as the hub and bottleneck components in the platinum resistance-associated miRNA-mRNA network (Fig. 1a), further supporting their strong regulatory ability.

**Table 1:** Details of the platinum resistance-associated miRNA biomarkers identified in this study

miRNA ID	Log_2_(FC)	*P*-value	NSR^a^ value	*P*-value of NSR	NPRG^b^ value
miR-454-3p	1.50	0.0228	33	2.98E−08	6
miR-98-5p	0.93	0.0165	24	5.96E−08	3
miR-183-5p	0.81	0.0441	5	0.001048	1
miR-22-3p	1.00	0.0406	4	0.001988	1

^a^Number of single-line regulation in the miRNA-mRNA network

^b^Number of platinum resistance-associated genes (NPRG) among miRNA targets

### Prognostic performance of the identified miRNA biomarkers

**Fig. 3:**
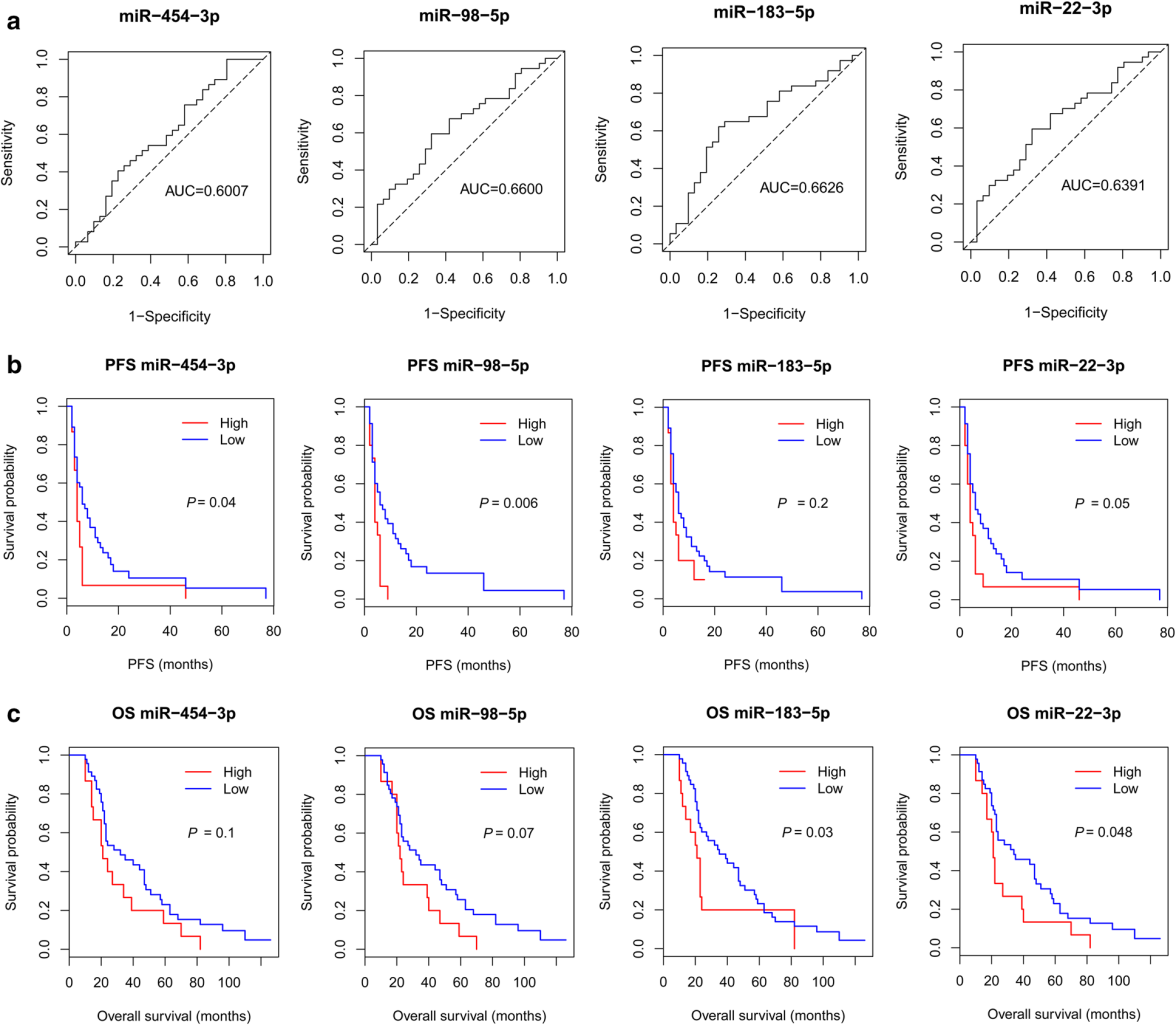
Prognostic performance of the identified miRNA biomarkers evaluated based on ROC curve and Kaplan–Meier survival analyses. **a** Sensitivity and specificity of miRNA biomarkers in predicting platinum resistance in HGSC were assessed by ROC curve analysis and AUC values. The dashed diagonal line in the ROC plot refers to AUC = 0.5, which means discrimination with random chance. **b**, **c** Kaplan–Meier survival curve analyses for progression-free survival (PFS) (**b**) and overall survival (OS) (**c**) of HGSC patients were conducted to evaluate the prognostic performance of miRNA biomarkers in predicting survival times. *P*-values were calculated using the log-rank test

To evaluate the performance of the platinum resistance-associated miRNA biomarkers, ROC curve analysis was performed to evaluate sensitivity and specificity of these miRNA biomarkers in distinguishing between platinum-sensitive and platinum-resistant HGSC patients. As shown in Fig. 3a, the area under curve (AUC) values ranged from 0.60 to 0.66, indicating relatively superior prediction performance of the identified miRNA biomarkers.

Kaplan–Meier survival analysis of HGSC patients with relatively high- or low-level expression patterns of miRNA biomarker was further conducted to measure the ability of miRNA biomarkers in predicting clinical outcomes. All four biomarkers tended to be associated with PFS and OS times of patients with HGSC. Notably, patients with high expression level of miR-454-3p (*P *= 0.04, log-rank test) and miR-98-5p (*P *= 0.006, log-rank test) had a significantly shorter PFS time, while those patients with high expression level of miR-183-3p (*P *= 0.03, log-rank test) and miR-22-3p (*P *= 0.048, log-rank test) had a significantly shorter OS time (Fig. 3b, c). Thus, the results revealed that miR-454-3p and miR-98-5p had prognostic potential in predicting progression outcome, while miR-183-3p and miR-22-3p may serve as prognostic biomarkers in evaluating overall clinical outcome.

### Functional roles of the identified miRNA biomarkers associated with platinum resistance

**Fig. 4:**
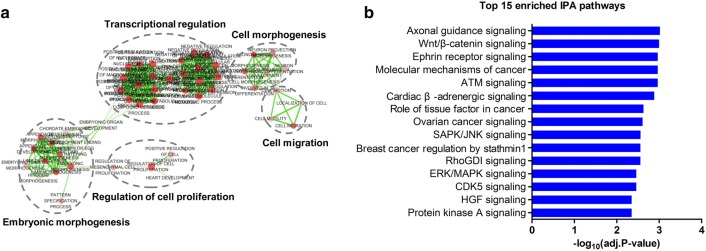
Gene ontology (GO) and pathway enrichment analyses of target genes of the identified miRNA biomarkers. **a** Clusters of miRNA biomarker target-enriched GO terms at the biological process level. In the functional enrichment map, each node refers to a GO term and is grouped based on GO similarity; each edge represents there were shared genes between two connecting GO terms. Node size is determined by the gene number in each GO term. Node color intensity represents enrichment significance. Edge thickness is determined by the number of shared genes between two connecting GO terms. **b** Top 15 significantly enriched IPA pathways of target genes of the identified miRNA biomarkers

To gain further insights into the putative biological roles of the four identified miRNA biomarkers, DAVID was employed for enrichment analyses of their target genes based on GO terms. As shown in Fig. 4a, significantly enriched GO terms at the biological process level were mainly clustered into five groups (transcriptional regulation, embryonic morphogenesis, cell morphogenesis, cell migration and regulation of cell proliferation), which were closely linked to platinum resistance in ovarian cancer. Accumulating evidence has demonstrated that dysregulation of extensive genes expression is a hallmark of cancer cells [30], and transcriptional regulation mediated by miRNAs contributes to chemoresistance in ovarian cancer [13, 33]. Furthermore, the epithelial-mesenchymal transition (EMT), an important step in morphogenesis, is associated with cancer progression and metastasis [34]. Matassa and co-workers disclosed that down-regulation of TRAP1 promotes cell migration and EMT [35] along with inducing resistance to cisplatin-based chemotherapy [36].

**Fig. 5:**
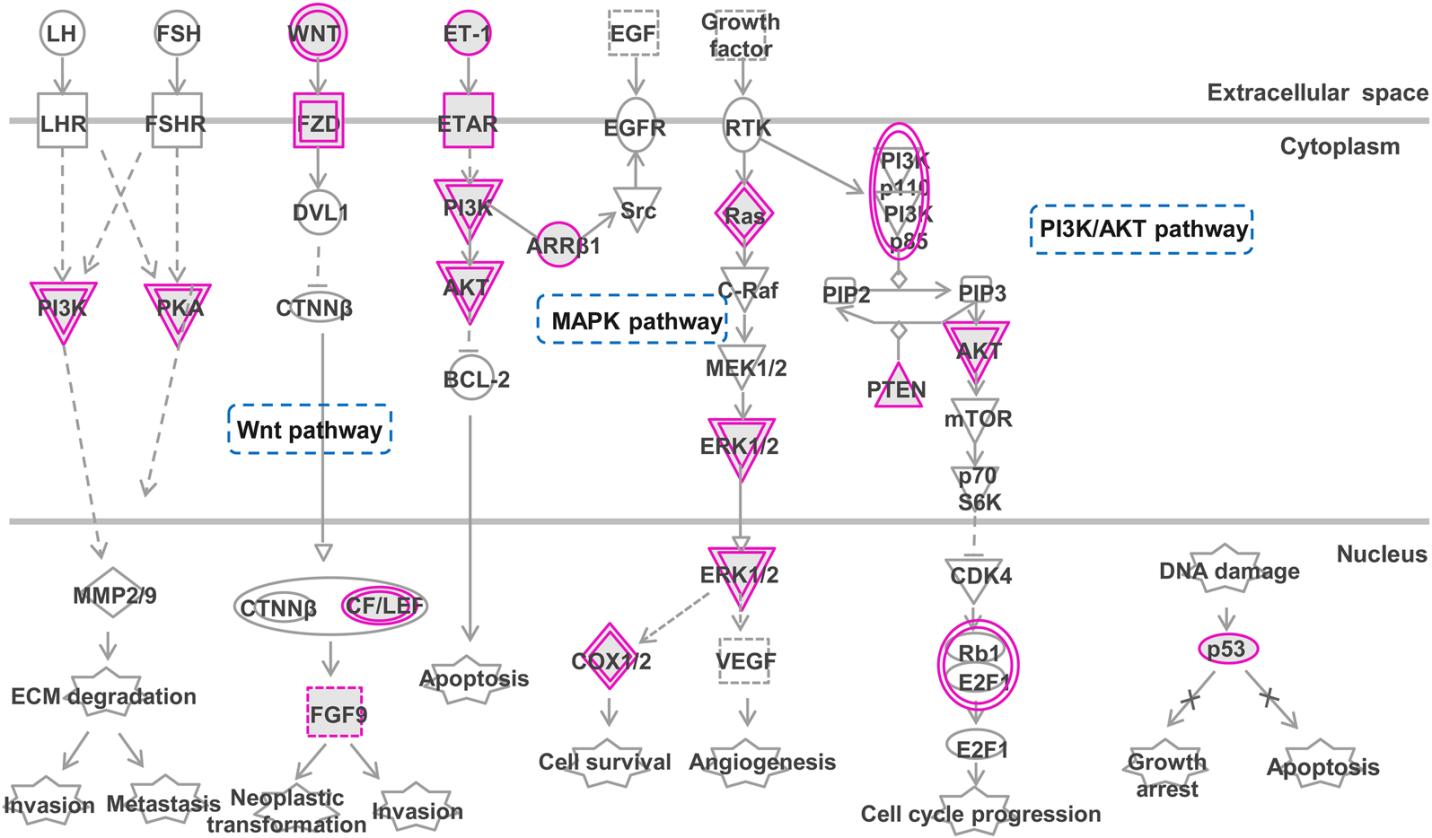
The ovarian cancer pathway enriched by target genes of the identified miRNA biomarkers in IPA. Objects with purple circles or triangles are acting locus by mapped genes

We further performed functional enrichment analysis on the target genes of miRNA biomarkers based on IPA pathways. Here, we focused on the top 15 enriched signaling pathways, e.g., axon guidance signaling, Wnt/β-catenin signaling, ephrin receptor signaling, ATM signaling, ovarian cancer signaling, and SAPK/JNK signaling (Fig. 4b). Overall, target genes of the identified miRNA biomarkers enriched in ovarian cancer signaling were closely linked with Wnt/β-catenin, MAPK and PI3 K/AKT pathways that mediate tumor invasion and metastasis, cell survival and apoptosis (Fig. 5, Additional file 6). Given the critical role of Wnt/β-catenin signaling in stem cell development [37], it has been found to be implicated in initiation and progression of multiple cancer types including ovarian cancer via components mediating cell proliferation and apoptosis [38, 39]. Upregulation of the Wnt pathway has been shown to promote chemoresistance in ovarian cancer via modulating cellular stem-like properties [40] or EMT [41]. Strikingly, axon guidance signaling pathway that closely associated with various types of cancers, such as prostate cancer [42] and pancreatic cancer [43], was the most enriched pathway of the target genes of miRNA biomarkers (Additional file 7). Increasing evidence shows that many proteins of axon guidance pathway play critical roles in tumorigenesis [44]. As a target gene of miR-454-3p, EFNA5 is an important component of axon guidance pathway and has been recognized as promising prognostic biomarker and therapeutic target for ovarian cancer [45]. In addition, the ATM signaling (Additional file 8) and SAPK/JNK signaling (Additional file 9) enriched by target genes of the identified miRNA biomarkers were associated with chemoresistance by mediating DNA damage and repair, and apoptosis, respectively [46, 47].

Therefore, we have uncovered that the miRNA biomarkers could serve as key regulators by targeting genes implicated in tumor invasion and metastasis, cell cycle, apoptosis, etc., thereby playing critical roles in chemoresistance in ovarian cancer.

### Validation of the identified miRNA biomarkers in platinum-resistant and platinum-sensitive cell lines

**Fig. 6:**
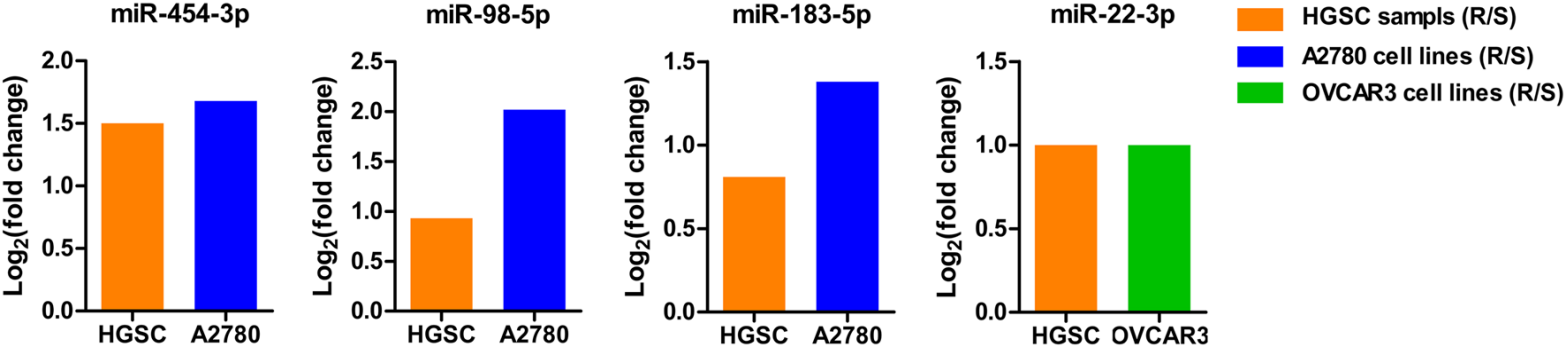
Validation of expression patterns of the identified miRNA biomarkers in A2780 and/or OVCAR3 ovarian cancer cells. The orange color represents the fold change of miRNAs between platinum-resistant and platinum-sensitive HGSC patients; the blue color represents the fold change of miRNAs between platinum-resistant and platinum-sensitive A2780 cell lines; the green color represents the fold change of miRNAs between platinum-resistant and platinum-sensitive OVCAR3 cell lines

To further validate association of the identified miRNA biomarkers with platinum resistance, their expression pattern between platinum-sensitive and platinum-resistant ovarian cancer cell lines were analyzed using miRNA expression datasets from GSE84200. Then, the log_2_(fold change) of miRNA biomarkers between platinum-resistant and platinum-sensitive HGSC tissue (termed as HGSC samples (R/S)) was compared with that between platinum-resistant and platinum-sensitive ovarian cancer cell line A2780 (termed as A2780 (R/S)) or OVCAR3 (termed as OVCAR3 (R/S)). As shown in Fig. 6, miR-454-3p, miR-98-5p and miR-183-5p had a consistent trend of expression alteration in HGSC tissue and A2780 cells, while miR-22-3p displayed the same fold change of expression level in HGSC tissue and OVCAR3 cells. Therefore, the expression pattern of the four miRNA biomarkers was confirmed in ovarian cancer cell lines.

## Discussion

In this study, to gain insights into the mechanisms underlying chemoresistance in HGSC, an integrated method was proposed for identifying putative platinum resistance-associated miRNA biomarkers through constructing PRMNH that consists of regulatory miRNA-mRNA pairs with differential expression patterns between platinum-resistant and platinum-sensitive HGSC patients. Given the critical importance of vulnerable nodes in the biological network structure, this approach was improved by introducing the criteria of number of platinum resistance-associated genes (NPRG) among miRNA targets based on the theory of single-line regulation, which has been successfully applied to identify miRNA biomarkers for detection of pediatric acute myeloid leukemia and prostate cancer metastasis [15, 20]. Thus, comprehensive evaluation of miRNA structural vulnerability and functional relevance to chemoresistance would facilitate the identification of platinum resistance-associated miRNA biomarkers.

Based on the computational approach, four miRNAs (miRNA-454-3p, miRNA-98-5p, miR-183-5p and miR-22-3p) were identified as putative biomarkers for predicting platinum resistance in HGSC. The expression patterns of these miRNAs were further validated in ovarian cancer cell lines, A2780 or OVCAR3 (Fig. 6). Consistent with our results, Wang et al. [23] reported up-regulation of miR-98-5p in cisplatin-resistant epithelial ovarian cancer. Furthermore, they uncovered that increased miR-98-5p expression could inhibit miR-152 expression by directly regulating Dicer 1, thereby inducing cisplatin resistance in epithelial ovarian cancer [23]. Van et al. [48] demonstrated that miR-22 is up-regulated in platinum-resistant ovarian cancer patients. Another study by Zhou et al. [49] revealed that miR-22 overexpression results in not only suppression of proliferation and migration, but also increased cisplatin sensitivity of osteosarcoma cells. Zhao et al. [50] found that miR-454-3p-mediated ceRNA interaction between lncRNA HOXA11-AS and Stat3 could promote cisplatin resistance of lung adenocarcinoma cells. Up-regulation of miR-183-5p has been reported in multiple cancer types including ovarian cancer [51], endometrial cancer [52] and lung cancer [53]. However, no evidence on the potential contribution of miR-454-3p and miR-183-5p to platinum resistance in ovarian cancer has been documented. Therefore, miR-454-3p and miR-183-5p were firstly discovered to be associated with chemoresistance in ovarian cancer, highlighting the role of the present study in broadening our understanding of miRNAs involved in platinum resistance.

It’s well known that prognosis assessment is of great significance for making an appropriate therapeutic strategy. Due to tumor heterogeneity [54], we observed apparent individual differences of miRNA and mRNA expression patterns either in the platinum-sensitive group or in the platinum-resistant group. Accordingly, the AUC values of the four miRNA biomarkers ranged from 0.60 to 0.66, indicating relatively higher predictive capability for stratifying platinum-sensitive and platinum-resistant HGSC patients. Furthermore, the four miRNA biomarkers served as independent predictive factors of PFS or OS in HGSC patients, implying good prognostic performance. Thus, our method, mainly based on the miRNA-mRNA regulatory network and single-line regulation theory, was robust in identifying platinum resistance-associated miRNA signatures in HGSC.

Efforts have been made in the present study to explore the functional mechanism of the identified miRNA biomarkers by GO and IPA pathway enrichment analysis of their target genes. Notably, target genes of the four miRNA biomarkers were closely implicated in cancer progression-related processes, such as transcriptional regulation, morphogenesis, and cell migration and proliferation (Fig. 4a). Platinum drugs function by forming DNA-platinum adducts, leading to DNA damage and further inducing apoptosis [4]. Target genes were further shown to be enriched in platinum resistance-associated biological pathways, including axon guidance signaling related to tumorigenesis, Wnt/β-catenin signaling associated with cell proliferation and apoptosis, ATM signaling implicated in DNA damage and repair, SAPK/JNK signaling related to apoptosis, RhoGDI signaling implicated in tumor proliferation and metastasis and CDK5 signaling involved in cell cycle, etc (Fig. 4b). Our collective findings clearly suggested that the identified miRNA biomarkers contributed to platinum resistance in HGSC by regulating cell proliferation, migration, apoptosis and cell cycle. Thus, the identified miRNA biomarkers indicating platinum resistance could serve as potential predictors of therapeutic response for HGSC patients, thereby promoting clinical improvements in management and therapy for HGSC.

## Conclusion

In summary, by employing a network-based computational method, we discovered four miRNA biomarkers, miR-454-3p, miR-98-5p, miR-183-5p and miR-22-3p that could potentially serve as indicators of resistance to platinum-based chemotherapy thereby contributing to reduce treatment costs and improve patients’ prognosis. The functions of the identified miRNA biomarkers were further explored via GO and IPA pathway enrichment analyses, providing insights into the mechanisms by which these miRNAs contribute to platinum resistance in HGSC.

## Supplementary information

**Additional file 1.** Differentially expressed miRNAs between platinum-resistant and platinum-sensitive HGSC patients.

**Additional file 2.** Differentially expressed mRNAs between platinum-resistant and platinum-sensitive HGSC patients.

**Additional file 3.** The PRMNH composed by differentially expressed miRNAs and mRNAs.

**Additional file 4.** Known miRNAs associated with platinum resistance in ovarian cancer.

**Additional file 5.** The feature of target genes of the identified miRNA biomarkers in PRMNH.

**Additional file 6.** The Wnt signaling pathway enriched by targets of the identified miRNA biomarkers in IPA. Objects with purple circles or triangles were acting locus by mapped genes.

**Additional file 7.** The axon guidance signaling pathway enriched by targets of the identified miRNA biomarkers in IPA. Objects with purple circles or triangles were acting locus by mapped genes.

**Additional file 8.** The ATM signaling pathway enriched by targets of the identified miRNA biomarkers in IPA. Objects with purple circles or triangles were acting locus by mapped genes.

**Additional file 9.** The SAPK/JNK signaling pathway enriched by targets of the identified miRNA biomarkers in IPA. Objects with purple circles or triangles were acting locus by mapped genes.

## Data Availability

The miRNA data in the current study are based on public data available in the Gene Expression Omnibus (GEO: http://www.ncbi.nlm.nih.gov/geo/) with the accession number GSE65821. Transcriptome sequencing data in the current study are based on public data available in the European Genome-phenome Archive (EGA) repository under the accession code EGAD00001000877. The processed miRNA and mRNA expression data of 31 platinum-sensitive and 37 platinum-resistant HGSC patients were downloaded from the publicly available International Cancer Genome Consortium (ICGC) based on search term ‘OV-AU’.

## Abbreviations

HGSC: high-grade serous ovarian cancer
miRNA: microRNA
POMA: Pipeline of Outlier MicroRNA Analysis
MicroRNA-BD: MicroRNA Biomarker Discovery
ICGC: International Cancer Genome Consortium
GEO: Gene Expression Omnibus
EGA: European Genome-phenome Archive
eBayes: empirical bayes
PRMNH: platinum-resistance associated miRNA-mRNA network in HGSC
BC: betweenness centrality
CC: closeness centrality
NSR: number of single-line regulation in the miRNA-mRNA network
NPRG: number of platinum resistance-associated genes
ROC: receiver operating characteristic
AUC: area under curve
PFS: progression free survival
OS: overall survival
IPA: Ingenuity Pathway Analysis
EMT: epithelial-mesenchymal transition
