# Supplementary figures and images for: Network vulnerability-based and knowledge-guided identification of microRNA biomarkers indicating platinum resistance in high-grade serous ovarian cancer
Source: Clin Transl Med. 2019 Oct 29;8:28. doi: 10.1186/s40169-019-0245-6 (PMC6820656; doi:10.1186/s40169-019-0245-6)

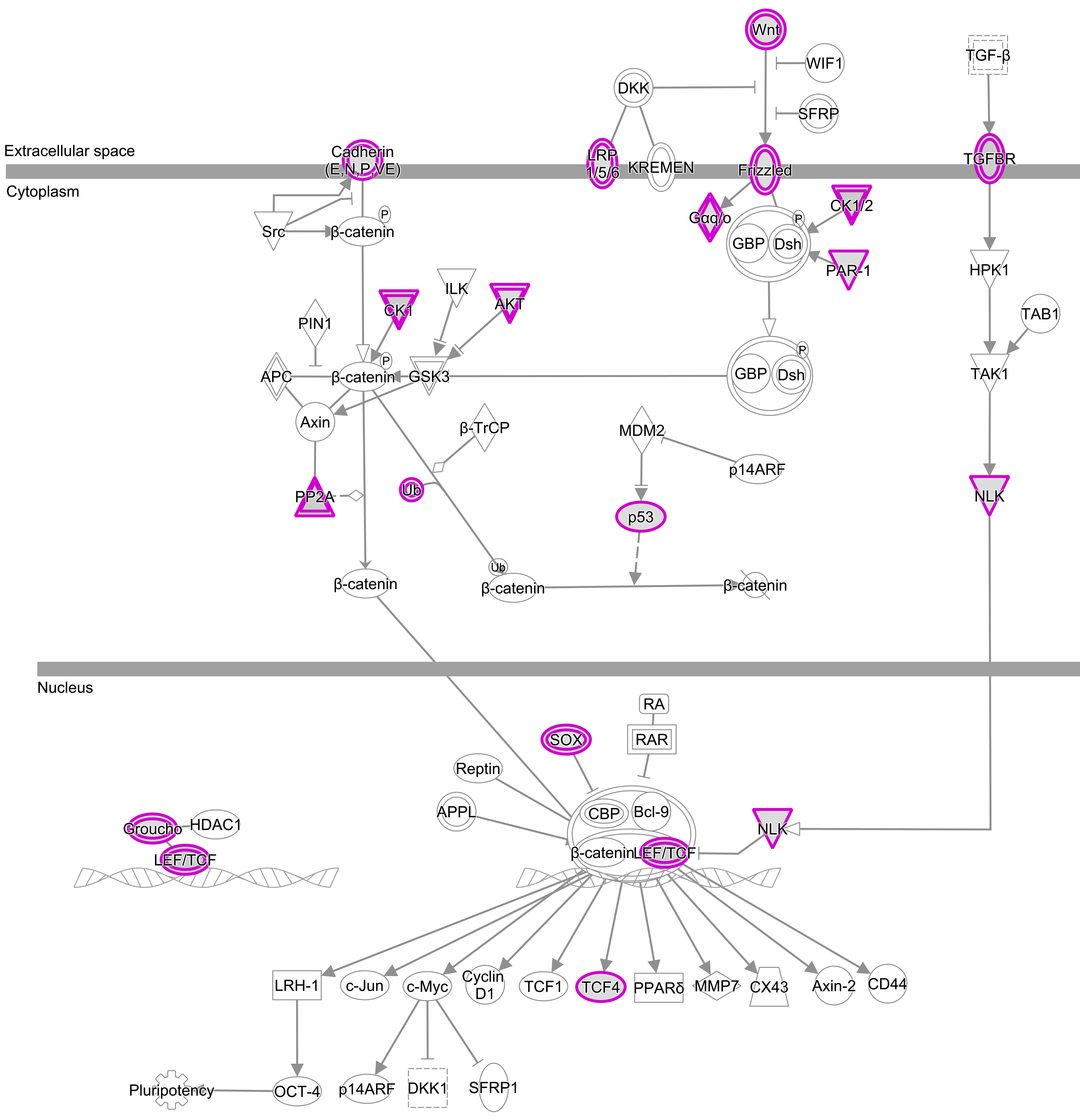

Supplement: Supplementary file 6 — Additional file 6. The Wnt signaling pathway enriched by targets of the identified miRNA biomarkers in IPA. Objects with purple circles or triangles were acting locus by mapped genes. [file 40169_2019_245_MOESM6_ESM.tif]

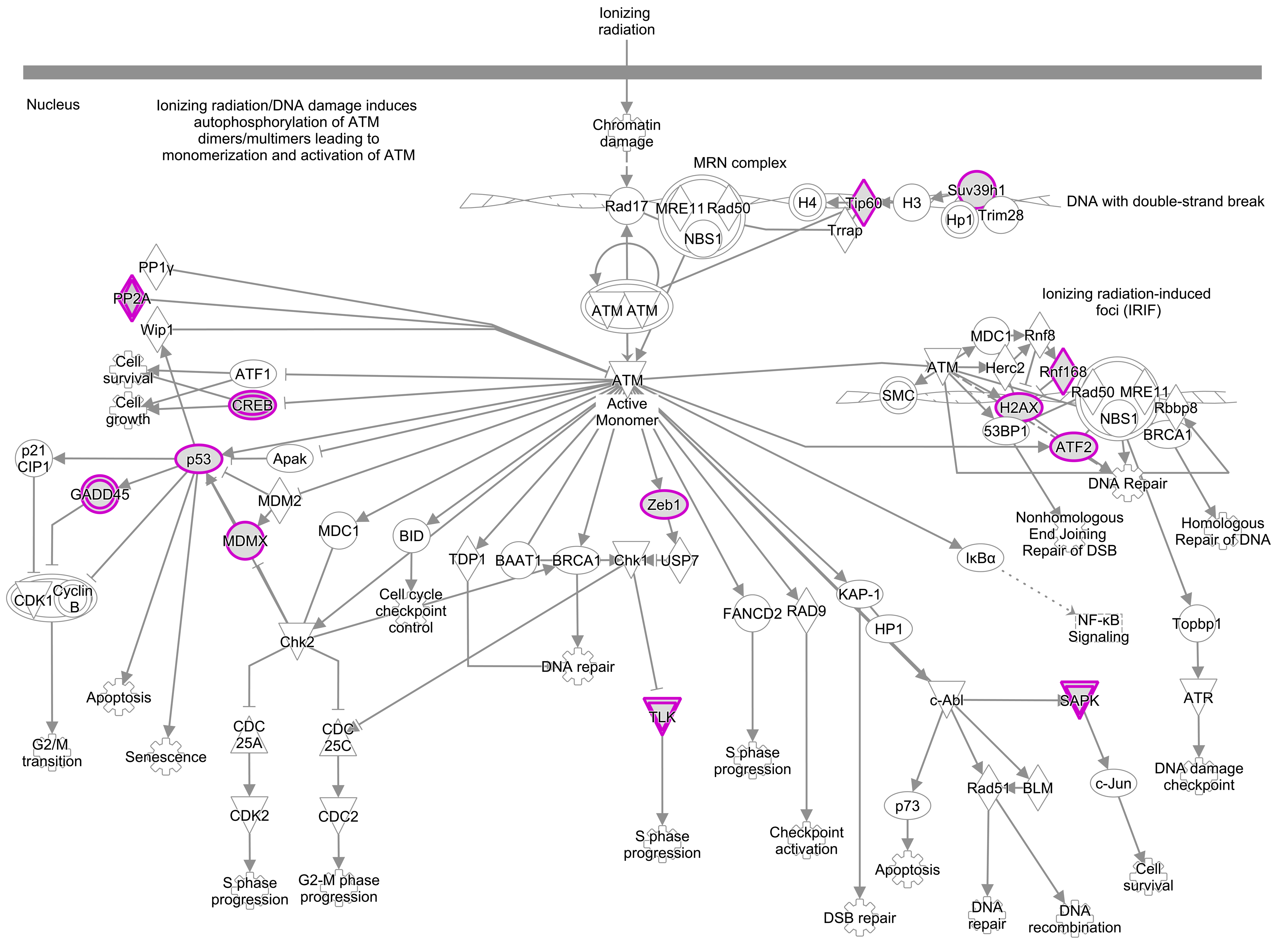

Supplement: Supplementary file 8 — Additional file 8. The ATM signaling pathway enriched by targets of the identified miRNA biomarkers in IPA. Objects with purple circles or triangles were acting locus by mapped genes. [file 40169_2019_245_MOESM8_ESM.tif]

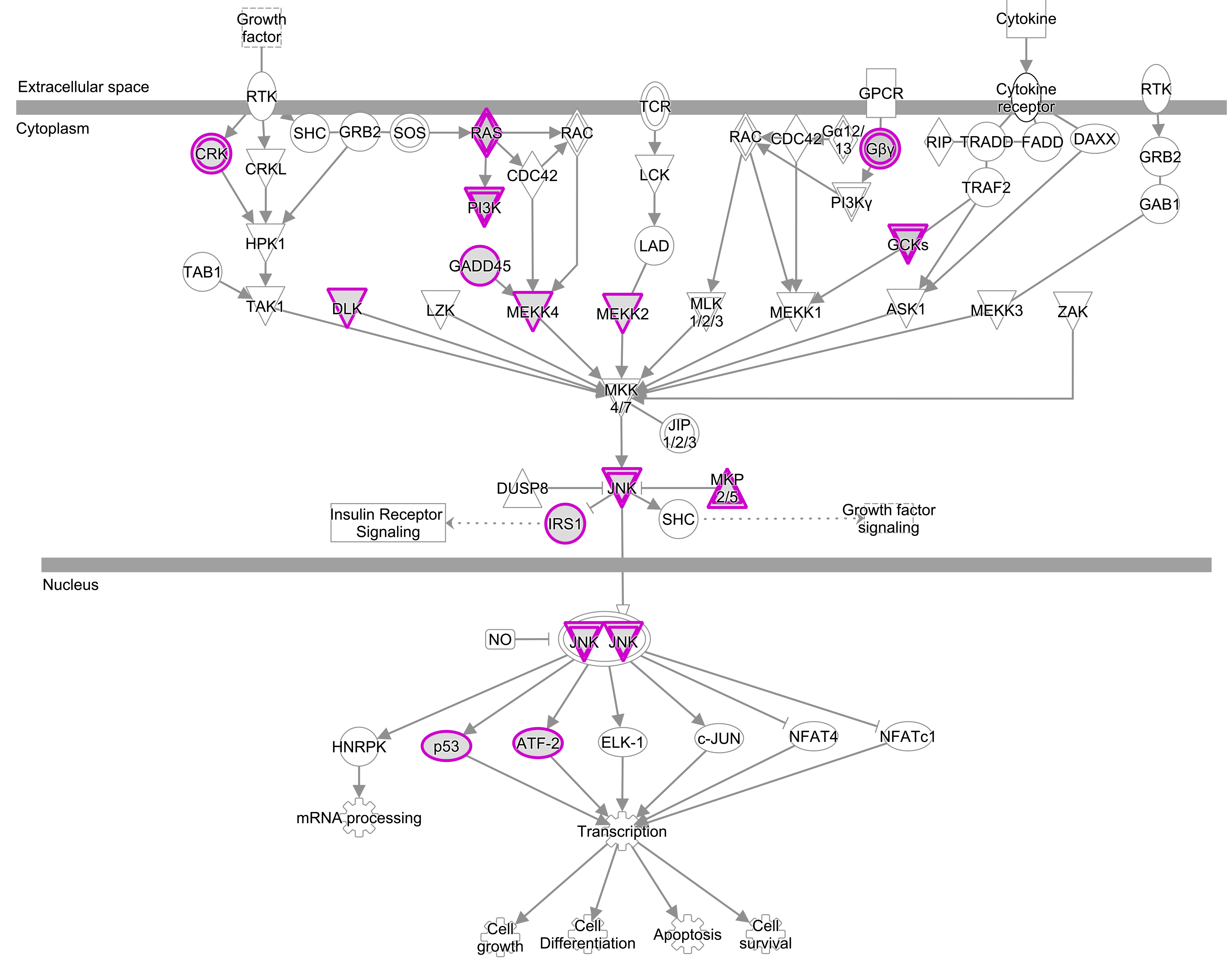

Supplement: Supplementary file 9 — Additional file 9. The SAPK/JNK signaling pathway enriched by targets of the identified miRNA biomarkers in IPA. Objects with purple circles or triangles were acting locus by mapped genes. [file 40169_2019_245_MOESM9_ESM.tif]
